# Coronatine Facilitates *Pseudomonas syringae* Infection of *Arabidopsis* Leaves at Night

**DOI:** 10.3389/fpls.2016.00880

**Published:** 2016-06-21

**Authors:** Shweta Panchal, Debanjana Roy, Reejana Chitrakar, Lenore Price, Zachary S. Breitbach, Daniel W. Armstrong, Maeli Melotto

**Affiliations:** ^1^Department of Biology, University of Texas at ArlingtonArlington, TX, USA; ^2^Department of Plant Sciences, University of California, DavisDavis, CA, USA; ^3^Department of Chemistry, University of Texas at ArlingtonArlington, TX, USA

**Keywords:** coronatine biosynthesis, pathogen penetration, stomatal immunity, phytotoxin, plant defense

## Abstract

In many land plants, the stomatal pore opens during the day and closes during the night. Thus, periods of darkness could be effective in decreasing pathogen penetration into leaves through stomata, the primary sites for infection by many pathogens. *Pseudomonas syringae* pv. *tomato* (*Pst*) DC3000 produces coronatine (COR) and opens stomata, raising an intriguing question as to whether this is a virulence strategy to facilitate bacterial infection at night. In fact, we found that (a) biological concentration of COR is effective in opening dark-closed stomata of *Arabidopsis thaliana* leaves, (b) the COR defective mutant *Pst* DC3118 is less effective in infecting *Arabidopsis* in the dark than under light and this difference in infection is reduced with the wild type bacterium *Pst* DC3000, and (c) *cma*, a COR biosynthesis gene, is induced only when the bacterium is in contact with the leaf surface independent of the light conditions. These findings suggest that *Pst* DC3000 activates virulence factors at the pre-invasive phase of its life cycle to infect plants even when environmental conditions (such as darkness) favor stomatal immunity. This functional attribute of COR may provide epidemiological advantages for COR-producing bacteria on the leaf surface.

## Introduction

Plants are exposed to different types and combinations of environmental conditions every day. For instance, daily fluctuations in temperature, light conditions, humidity, CO_2_ concentration, water availability, and UV exposure are common in nature. Being sessile organisms, plants rely on specific mechanisms that sense these changes in environmental conditions and relay the message to plant cells, leading to adaptation to those conditions for optimum growth and reproduction. This is extremely crucial in case of crop plants, where yields are largely dependent on environmental conditions. Along with abiotic factors affecting plant productivity, plants are continuously and simultaneously exposed to biotic stresses. Several species of bacteria, fungi, viruses, and nematodes can cause infections in addition to insects that can act as herbivores. To ward off pathogens, plants possess an innate immune system, which recognizes pathogens and sets off immunity weapons. In the field, biotic and abiotic stressors often occur together and can have a positive or negative combinatory impact on the plant (Suzuki et al., 2014). For example, cold and heat stress leads to lower resistance of plant to viruses (Szittya et al., 2003). It has also been shown that plant responses to such combinations of stimuli are more complicated and different than the response to a single stimulus in isolation (Suzuki et al., 2014). Thus, it becomes crucial to study combinations of abiotic and biotic factors to better understand plant responses in natural conditions.

One abiotic factor with major impact on plant growth and survival is light. Apart from the obvious use in photosynthesis, differences in light conditions can also affect plant defenses. For instance, shading increases infection by a range of pathogens (Roberts and Paul, 2006) most likely due to increased leaf surface wetness in the shade that favors pathogen fitness (Hirano and Upper, 2000) and/or inactivation of jasmonate-mediated plant defenses (Chico et al., 2014). In addition to quantity, the quality of light (e.g., white, red, and blue) may regulate bacterial behavior and virulence as has been observed for *Pseudomonas syringae* pv. *tomato* DC3000 (*Pst* DC3000) on tomato leaves (Río-Álvarez et al., 2014). Thus, daily light cycles may alter the physiology of both plants and microbes affecting the outcome of plant–pathogen interactions.

Sensing darkness influences both the activation of plant response and the circadian rhythm. Plant’s innate circadian rhythm that allows for regular photosynthesis and food production also controls guard cells movement. Stomata of C3 and C4 plants are open during the day and closed during the night. Apart from the basic function of exchange of gases and regulation of water loss (Zelitch, 1969; Schroeder et al., 2001; Fan et al., 2004), stomata are the port of entry for microbial invasion in the leaf tissue (Agrios, 2005). Thus, darkness may provide a direct prevention of pathogen infection by stomatal closure. In addition to closing stomatal pores in response to darkness, plants have evolved mechanisms to prevent pathogen entry by closing of stomatal pores even under bright light (McDonald and Cahill, 1999; Melotto et al., 2006; Gudesblat et al., 2009; Zhang et al., 2010; Hettenhausen et al., 2012; Roy et al., 2013). This phenomenon occurs by direct recognition of pathogen-associated molecular patterns (PAMP) by guard cells, which relays information to close the stoma (Lee et al., 1999; Klüsener et al., 2002; Melotto et al., 2006; Zeng and He, 2010; Casabuono et al., 2011). The presence of pathogens during the day thus provides two opposing factors for guard cell movement. Pathogen-triggered stomatal closure during the day, however, depends on the strength of the PAMP signaling (Roy et al., 2013).

The stomatal response to an individual factor is not always the same as when it is exposed to a combination of factors (Merilo et al., 2014). For example, a combination of virus and drought, virus and heat, and all three together leads to closed stomata. However, only heat stress or only virus infection leads to open stomata (Prasch and Sonnewald, 2013). In guard cells, an intricate network of signaling pathways is involved in opening and closing of stomata in response to biotic and abiotic stresses, including ion fluxes, sugar transport, cytoskeleton rearrangement, hormone signaling, and regulation of gene expression (Schroeder et al., 2001; Montillet and Hirt, 2013). However, how guard cells sense and prioritize their response to multiple, simultaneous signals still remains elusive.

Several pathogen virulence factors can open PAMP-closed stomata (Melotto et al., 2008; McLachlan et al., 2014) such as the fungal fusiccocin (Squire and Mansfield, 1974), *Xanthomonas campestris* DSF (Gudesblat et al., 2009), and *Pseudomonas syringae* syringolin A (Schellenberg et al., 2010) and coronatine (COR; Melotto et al., 2006). COR is one of the most well studied phytotoxins that acts on both pre- and post-invasive stages of *P. syringae* life cycle (Melotto and Kunkel, 2013). COR-producing strains of *P. syringae* have been found to be more aggressive than the COR-defective mutants (Brooks et al., 2004; Ishiga et al., 2009). COR is a structural and functional mimic of the plant hormone jasmonoyl isoleucine (JA-Ile; Zhao et al., 2003; Katsir et al., 2008; Melotto et al., 2008). JA-Ile is a lipid-derived hormone with regulatory functions in vegetative and reproductive growth, defense responses against abiotic stresses such as ultraviolet light and ozone, insect herbivory, and necrotrophic pathogens (Katsir et al., 2008). COR activates JA signaling, induces JA-responsive genes in *Arabidopsis*, and contributes to disease development by antagonizing salicylic acid (SA) signaling, a plant hormone actively involved in plant defense against *P. syringae* (Glazebrook et al., 2003; Uppalapati et al., 2007). The mode of action of COR and JA in the plant cell has been the subject of intensive research as discussed in recent reviews (Melotto et al., 2008; Pauwels and Goossens, 2011).

In this study, we focused on elucidating the effectiveness of COR in overcoming stomatal immunity and promoting bacterial infection at night. Here, we demonstrate that periods of darkness, which promotes stomatal closure in *Arabidopsis*, are effective in reducing bacterial penetration through stomata. We also provide evidence that COR biosynthesis is up-regulated in the epiphytic population of *Pst* DC3000 prior to its penetration into leaves, and COR opens dark-closed stomata at biologically relevant concentrations. These results suggest that production of COR provides a significant epidemiological advantage for *P. syringae* with major implications in plant infection by foliar bacteria even when environmental conditions favor stomatal immunity.

## Materials and Methods

### Plant Material and Growth Conditions

*Arabidopsis thaliana* (L. Heyhn.) ecotype Columbia (Col-0, ABRC stock CS60000) seeds were sown in a 1:1:1 v:v:v mixture of growing medium (Redi-earth plug and seedling mix, Sun Gro), fine vermiculite, and perlite (Hummert International, Earth City, MO, USA). Plants were grown in controlled environmental chambers at 22°C, 65 ± 5% relative humidity (RH), and a 12-h photoperiod under light intensity of 100 μmol.m^-2^.s^-1^. Four- to five-week old plants were used for all experiments.

### Bacterial Strains and Growth Conditions

Bacterium strains used were: *Pseudomonas syringae* pv. *tomato* DC3000 and its COR mutant DC3118 (gift from Sheng Yang He; Ma et al., 1991), KP105 (DC3000 wild type) and its COR mutant derivative *Pst* DB29 (gift from Carol Bender; Brooks et al., 2004), and *Pst* DC3000-pHW01 (a gift from Matthias Ullrich; Weingart et al., 2004). Bacterial cells were cultured in low-salt Luria–Bertani medium (Katagiri et al., 2002) at 30°C for all experiments. Medium was supplemented with the appropriated antibiotic: 100 μg.ml^-1^ rifampicin (all *Pst* strains used), 50 μg.ml^-1^ kanamycin (*Pst* DC3118 and *Pst* DB29), 25 μg.ml^-1^ chloramphenicol (*Pst* DC3000 pHW01).

### Stomatal Assay

For experiments starting with closed stomata, treatments were applied to the plants in the morning before the lights were turned on and plants were kept in the dark for the duration of the experiment. For experiments starting with open stomata, plants were kept under light for at least 3 h in the morning prior to applying treatment and maintained under light for the duration of the experiment.

For stomatal assays with purified chemicals, whole leaves were floated in water or 1.5 μM COR (Sigma–Aldrich, St. Louis, MO, USA) as recommended by Zhao et al. (2003) and incubated under 22°C, light intensity of 100 μmol.m^-2^.s^-1^ and 60 ± 5% RH.

Stomatal assays with epidermal peels or intact leaves were performed as previously described (Melotto et al., 2006; Chitrakar and Melotto, 2010), except that leaves were directly imaged without propidium iodide staining. Stomatal aperture width was measured with a laser scanning confocal microscope (LSM 510 Meta, Carl Zeiss Inc., Thornwood, NY, USA) or a Nikon Eclipse 80i fluorescent microscope equipped with DIC and long distance objectives (Nikon Corporations, Shinagawa-ku, Tokyo, Japan) to avoid the use of cover slip. All experiments were completed by 2 pm.

### Bacterial Pathogenesis Assay

*Arabidopsis* plants were acclimated under 65 ± 5% RH at 25°C for 12 h. The level of humidity was monitored with a digital hygrometer (Traceable^®^, VWR). Plants were dip-inoculated in the morning before the lights were turned on and after being in the dark for 12 h. A set of plants was kept under complete darkness for the duration of the experiment and another set was transferred to a 12 h daily light cycle right after inoculation for direct comparison with the dark treatment.

To prepare the inoculum, bacteria were cultured as described above until an OD_600_ of 0.8 was reached. Bacterial cells were collected by centrifugation and re-suspended in water to the final concentration of 1 × 10^8^ CFU.ml^-1^ containing 0.02% Silwet L-77 (Lehle seeds Co., Round Rock, TX, USA) for dip-inoculated plants or 1 × 10^6^ CFU.ml^-1^ containing 0.004% Silwet L-77 for vacuum-infiltrated plants. Inoculated plants were immediately incubated under the following conditions: 25°C, 65 ± 5% relative humidity, and 12 h of daily light (100 μmol.m^-2^.sec^-1^) or darkness for the duration of the experiment. At the indicated time points, leaves were surface-sterilized with 70% ethanol for 2 min. Bacterial population in the plant apoplast was determined using the serial dilution method as previously described (Katagiri et al., 2002). The experiment was repeated three times.

### *cma* Promoter Activity Assay

The *Pst* DC3000 (pHW01) strain that carries a construct with the *egfp* gene driven by the *cma* promoter was used for this assay. The abaxial side of intact *Arabidopsis* leaves was placed in contact with a *Pst* DC3000 (pHW01) suspension of 1 × 10^8^ CFU.ml^-1^ on a microscope slide and incubated in constant white light (80–90 μmol.m^-2^.s^-1^) or constant darkness at 22°C. Leaf petioles were never in contact with the bacterial suspension to prevent the induction of the *cma* promoter in the presence of leaf exudate. At different time points, the abaxial surface of the leaf was washed in sterile, distilled water and imaged under fluorescent microscope in search for attached epiphytic green bacterial cells to determine the timing of the *cma* promoter activation. Additionally, 5 μL of the bacterial inoculum in contact with leaf samples was imaged on a microscopic slide to determine whether attachment to the leaf surface is required for the *cma* promoter activation. Bacterial suspension not in contact with leaves was used as a control to determine whether contact with leaf surface is necessary for *cma* promoter activity and whether cells were still viable for the duration of the experiment (24 h). Micrographs were obtained and analyzed with a Nikon Eclipse 80i or a LSM 510 Meta microscope equipped with a GFP filter set and associated software.

### Coronatine Production *In Vitro* Assay

*Pst* DC3000 was grown in low sodium Luria–Bertani broth overnight and 1 × 10^8^ cells from this culture were transferred to liquid HSC medium (nutrients per liter: 1.0 g NH_4_Cl, 0.2 g MgSO_4_.7H_2_O, 4.1 g KH_2_PO_4_, 3.6 g K_2_HPO_4_.3H_2_O, 0.3 g KNO_3_, 10 mL of 2 mM FeCl_3_. Nine parts of this solution was amended with one part of 20% glucose) at 18°C for 24 h according to Palmer and Bender (1993). COR was extracted from the culture supernatant using the abbreviated extraction protocol as previously described (Palmer and Bender, 1993). Presence of COR was analyzed by HPLC on an ASTEC (Whippany, NJ, USA) C8 column (4.6 mm × 250 mm, 5 μm) at 208 nm. Isocratic separations were performed using a 0.05% trifluoroacetic acid/acetonitrile (60/40) mobile phase with a flow rate of 1.0 ml.min^-1^. The injection volume was 5 μl and the column temperature was 25°C. Calibration curves for COR were obtained with commercially available preparation (Sigma–Aldrich, St. Louis, MO, USA). The amount of COR produced was expressed as a function of protein concentration. The cells used for COR extraction were lysed by suspending in 1 M NaOH followed by boiling and freezing three times, and the protein content in bacterial cell lysates was determined with the Pierce BCA Protein Assay Kit (Thermo Fisher Scientific, Waltham, MA, USA).

### Statistical Analysis

Statistical significance of the results was calculated using ANOVA followed by Tukey–Kramer HSD at 95% confidence limit (InfoStat version 2012). All experiments reported here were repeated at least two times (biological replicates and a minimal of three technical replicates) with similar results.

## Results

### COR Prevents Bacterium-Triggered Stomatal Closure

**FIGURE 1:**
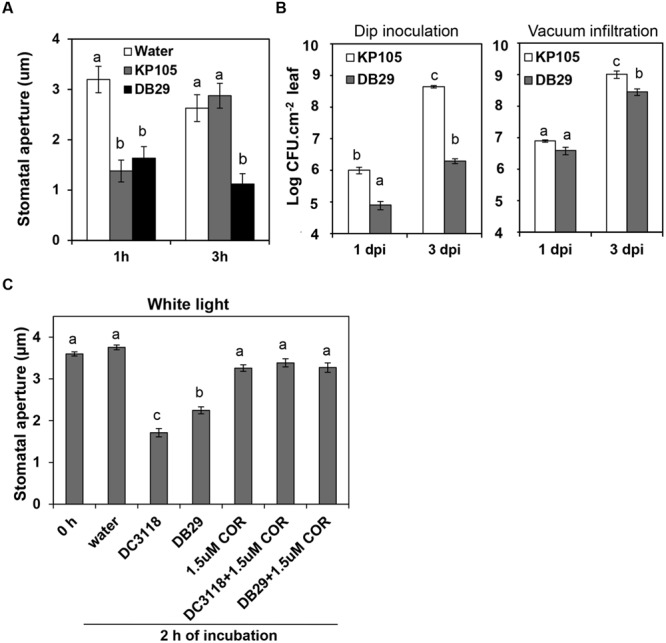
**Similar to *Pst* DC3118, *Pst* DB29 cannot overcome stomatal immunity and has reduced virulence on surface-inoculated plants. (A)** Stomatal aperture width in epidermal peels of Col-0 plants exposed to water, or *Pseudomonas syringae* pv. *tomato* (*Pst*) KP105 (DC3000 wild type parent) or *Pst* DB29 (COR defective mutant). **(B)** Bacterial enumeration in the apoplast of plants at 1 and 3 days after dip-inoculation (left graph) or vacuum-infiltration (right graph) with bacteria. Results are shown as the mean ± SE. Note that some error bars are too small to appear in the log scale graphs. Experiments were performed three times with similar results. **(C)** The graph shows stomatal aperture width in intact *Arabidopsis* leaves 2 h after dip-inoculation with bacterial suspension (*Pst* DC3118 and *Pst* DB29) with or without COR under light. Results in **(A)**, **(C)** are shown as the mean (*n* = 60) ± SE. Statistical significance (all panels) were detected with ANOVA followed by Tukey–Kramer HSD at 95% confidence limit.

We have previously shown that stomatal immunity leads to reduction of *P. syringae* infection of the leaf apoplast and COR can override this PAMP-triggered stomatal closure leading to bacterial penetration into the leaf tissue (Melotto et al., 2006). However, the use of a single bacterial mutant to support that conclusion raised the question as to whether the defects observed in these strains are due to the lack of COR production or due to pleiotropic effects of the mutation in the *Pst* DC3118 strain. Thus, we repeated the stomatal and pathogenesis assays with a second genetically characterized COR-mutant bacterium, *Pst* DB29 (Brooks et al., 2004). We found that *Pst* DB29 behaves similarly to *Pst* DC3118 in inducing a lasting stomatal closure (**Figure 1A**) as well as not being effective in colonizing the apoplast of surface-inoculated *Arabidopsis* plants (**Figure 1B**). In fact, the apoplastic population of the wild type bacterium *Pst* DC3000 (named KP105; Brooks et al., 2004) was 15- to 200-fold higher than the population of *Pst* DB29 at 1 and 3 days after surface inoculation, respectively. By contrast, twofold to fourfold difference between wild type and mutant populations was observed in vacuum-infiltrated plants at the same time points and significant difference was only observed at the third day (**Figure 1B**).

To further confirm that COR is the responsible and sufficient factor for *Pst* DC3000 to overcome bacterium-triggered stomatal closure (i.e., stomatal immunity), here we also show that addition of 1.5 μM COR to the inoculum was enough to complement *Pst* DC3118 and *Pst* DB29 to open the stomatal pore to the level of the water control (**Figure 1C**). As, we have shown before, COR alone does not cause any change in the stomatal aperture width under the light (**Figure 1C**).

Together, these results provide pharmacological and genetic evidence for the function of COR in overcoming stomatal immunity.

### Biological Concentration of COR Is Sufficient to Open Dark-Closed Stomata *In Vivo*

**FIGURE 2:**
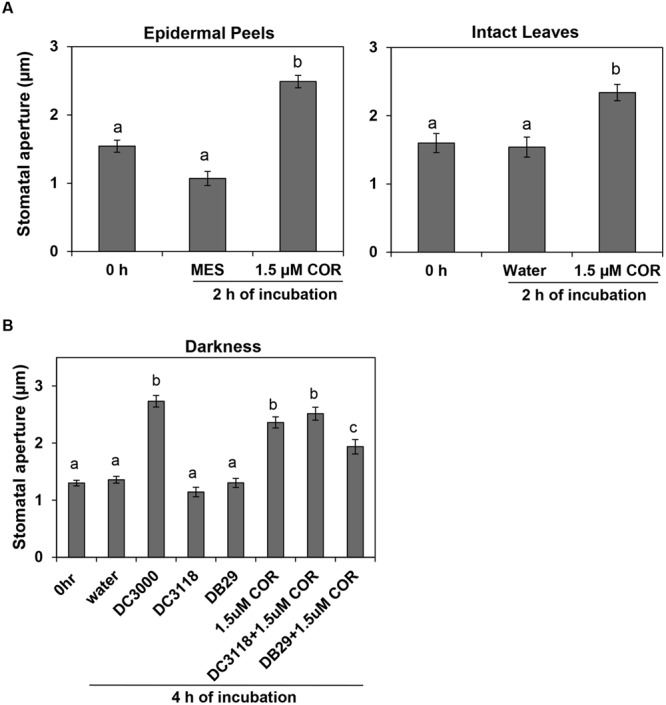
**Biological concentrations of coronatine (COR) induce stomatal opening and prevent stomatal closure. (A)** Stomatal aperture width of *Arabidopsis* epidermal peels (left) or leaves (right) incubated with purified COR under darkness. **(B)** The graph shows stomatal aperture width in intact *Arabidopsis* leaves 4 h after dip-inoculation with bacterial suspension (*Pst* DC3000, *Pst* DC3118, and *Pst* DB29) with or without COR in the dark. All results are shown as the mean (*n* = 60) ± SE. Statistical significance (all panels) were detected with ANOVA followed by Tukey–Kramer HSD at 95% confidence limit.

To address the possibility that COR action may support bacterial infection at night, we assessed whether COR could open dark-closed stomata. In fact, COR, at a concentration as low as 1.5 μM, was significantly effective in opening stomatal aperture in epidermal peels or intact leaves within 2 h of treatment (**Figure 2A**). The average width of the stomatal aperture increased drastically in tissue incubated with COR, whereas it remained the same in control tissues (**Figure 2A**). These results suggest that guard cells can directly perceive and respond to COR and this response may be independent of other tissue types as it was also observed in isolated epidermis.

To assess whether biological concentrations of COR produced during bacterial infection also could open dark-closed stomata, we dip-inoculated plants with the COR-producing *Pst* DC3000 and two COR-deficient mutants, *Pst* DC3118 and *Pst* DB29. While *Pst* DC3000 was able to open dark-closed stomata in intact leaves within 4 h of incubation under darkness, *Pst* DC3118 and *Pst* DB29 were unable to do so (**Figure 2B**). Furthermore, addition of 1.5 μM COR to the mutant bacterium inoculum restores the ability of the bacterium to open dark-closed stomata (**Figure 2B**).

### Production of COR Provides an Advantage for Bacterial Penetration through Stomata under Periods of Darkness

**FIGURE 3:**
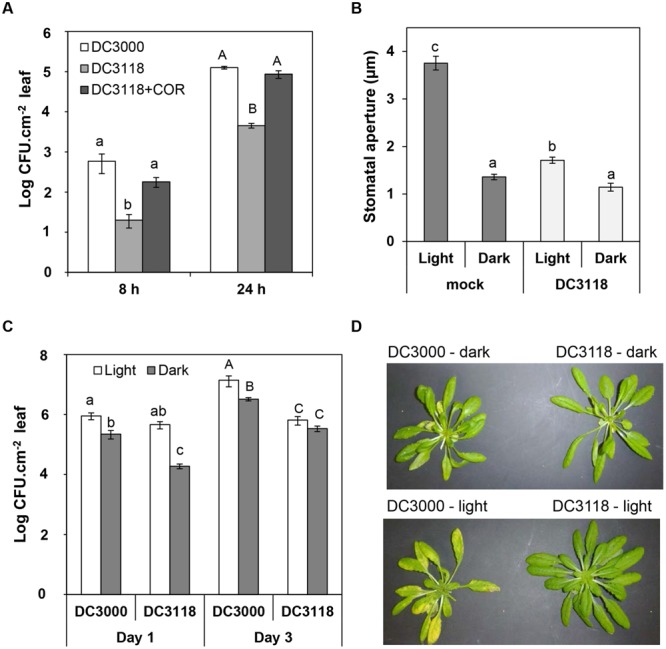
**COR provides advantage for *P. syringae* infection in the dark. (A)** Bacterial population in the plant apoplast of Col-0 plants dipped into a suspension (1 × 10^8^ CFU.ml^-1^) of *Pst* DC3000, *Pst* DC3118, or *Pst* DC3118 supplemented with 1.5 μM COR at 8 and 24 h after inoculation under complete darkness. **(B)** The graph shows stomatal aperture width in intact *Arabidopsis* leaves 2 h after dip-inoculation with *Pst* DC3118 or mock control under light or darkness. Results are shown as the mean (*n* = 60) ± SE. **(C)** Bacterial population in the plant apoplast of Col-0 plants dipped into a suspension (1 × 10^8^ CFU.ml^-1^) of *Pst* DC3000 or *Pst* DC3118 in the dark or under light at Day 1 and Day 3 after inoculation. Results in panels **(A,C)** are shown as mean of two biological replicates (*n* = 12) ± SE. Asterisks above the bars indicate statistical significance between the means within each time point (lower case letters = differences in the first time point; upper case letters = differences in the second time point). Statistical significance **(A**–**C)** were detected with ANOVA followed by Tukey–Kramer HSD at 95% confidence limit. **(D)** Symptoms were recorded 3 days after surface inoculation with the indicated bacteria and light conditions.

Next, we asked the question whether COR production represents an advantage for bacterial infection at night. We surface-inoculated *Pst* DC3118 with or without 1.5 μM COR in the dark and determined the size of the apoplastic bacterial population at early time points as an indirect measure of leaf penetration. At 8 and 24 h after inoculation, the *Pst* DC3118 population size was 20- to 25-fold larger when the inoculum was supplemented with COR as compared to the inoculum alone (**Figure 3A**). Furthermore, COR complemented the penetration defect of *Pst* DC3118 to the level of the wild type *Pst* DC3000 (**Figure 3A**).

Then, we reasoned that darkness significantly decreases leaf penetration by bacterium that cannot open stomata. If this hypothesis is true, apoplastic population of *Pst* DC3118 would be smaller in plants inoculated in the dark as compared to light in the first 24 h of infection (i.e., prior to extensive bacterial multiplication). First, we determined that simultaneous exposure of leaves to two stimuli, COR-mutant bacteria, and darkness, further decreased the stomatal aperture width as compared to that of leaves exposed to *Pst* DC3118 under light (**Figure 3B**). We also observed that exposing plants to darkness alone or darkness and *Pst* DC3118 leads to a similar average stomatal aperture width (**Figure 3B**) indicating that these two stimuli do not have an additive effect to close the stomatal pore and darkness is enough to induce an average stomatal closure to a maximum extent in this experimental setup. Roy et al. (2013) have also observed that incubation of *Salmonella enterica* on *Arabidopsis* leaves in the dark does not result into accentuated stomatal closure. Second, we surface-inoculated plants using the two light regimes side by side and counted apoplastic population of both wild type *Pst* DC3000 and the COR mutant *Pst* DC3118. The *Pst* DC3118 populations in the apoplast of plants under a 12 h light/12 h dark cycle was significantly larger than that of under complete darkness at 24 h post inoculation, but not at 3 days after inoculation (**Figure 3C**). The COR-producing *Pst* DC3000 was also able to infect and multiply aggressively in the apoplast, and it did so to a larger extent under light (**Figure 3C**). However, the difference in the *Pst* DC3000 populations between each light regime was reduced in comparison to *Pst* DC3118 populations.

As expected due to its reduced virulence, *Pst* DC3118 surface-inoculated plants showed no apparent symptoms after 3 days of surface-inoculation (**Figure 3D**). However, at this later stage of disease, *Pst* DC3000-infected plants showed less chlorosis in the dark as compared to plants kept at 12 h light/12 h dark cycle (**Figure 3D**), which correlated with a fourfold reduction in the leaf apoplastic bacterial population (**Figure 3C**).

### COR Biosynthesis Genes Are Activated on the Leaf Surface Independent of Light

Our previous studies showed that *Pst* DC3000 re-opens bacterium-closed stomata in a COR-dependent manner within 3 to 4 h of contact with plant tissue and we showed above that this bacterium also opens dark-closed stomata in the same time period (**Figure 2B**). These results raised an immediate question about the timing and location (i.e., epiphytic or endophytic) of COR production by *Pst* DC3000. To address this question, we used the reporter strain *Pst* DC3000 (pHW01) that contains a plasmid expressing green fluorescent protein (GFP) driven by the promoter of the COR biosynthesis gene *cma*. The appearance of green florescence over time indicates a high-level activation of the *cma* promoter, which is positively correlated with COR biosynthesis (Weingart et al., 2004).

**FIGURE 4:**
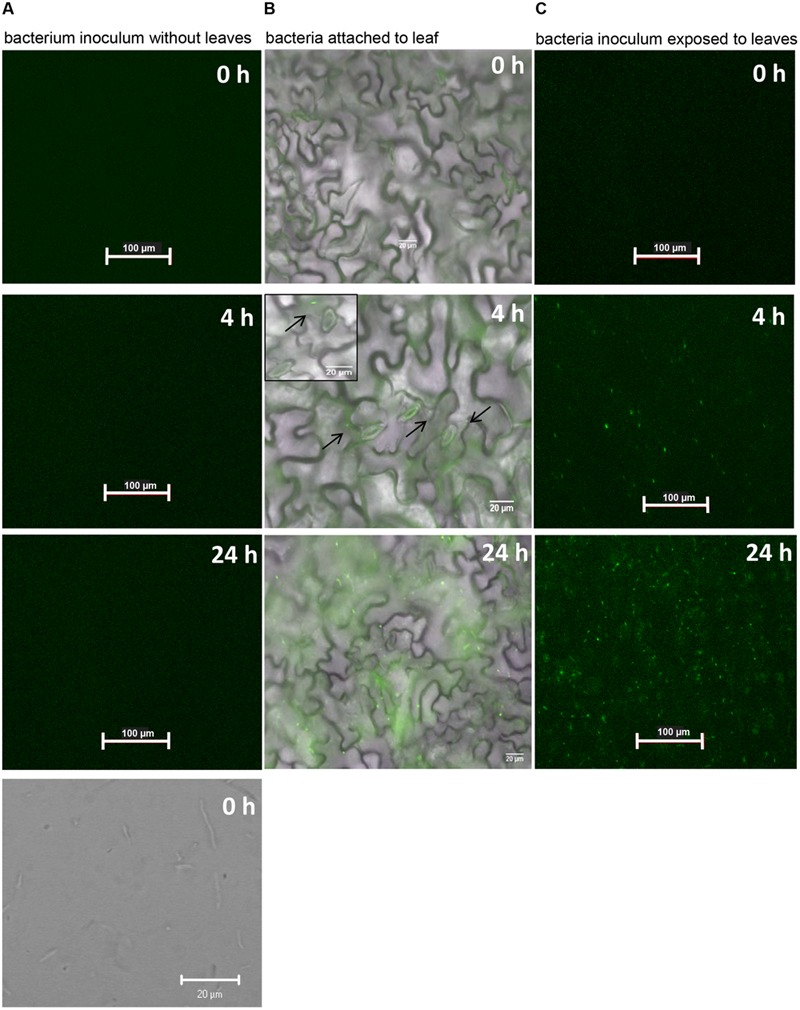
**COR biosynthesis reporter strain (*Pst* DC3000-pHW01) is induced in contact with the *Arabidopsis* leaf surface.** The pictures shown are representative of a time series fluorescence and bright field micrographs. **(A)** Micrographs of bacterial cell suspension showing that cells do not express GFP in the absence of leaf tissue. Top three pictures were taken with GFP filters and the bottom picture was taken under bright field. **(B)** Micrographs shows attached bacterial cells fluorescing after around 4 h of contact with the leaf surface. Black arrows on the middle micrograph show few fluorescing cells at 4 h. Insert on the middle micrograph shows a higher magnification that highlights well-defined glowing bacterial cells. **(C)** Micrographs show fluorescing bacterial cells in the inoculum exposed to the leaf surface.

Crude extracts and apoplastic fluids of tomato leaves have been shown to induce COR biosynthetic genes in *P. syringae* (Li et al., 1998). Thus, we used intact leaves to discard the possibility that the promoter was induced by the content of ripped mesophyll cells. We floated leaves on bacterial suspension and monitored the appearance of green fluorescent bacterial cells attached to the leaf surface or bacterial cells in suspension over a 24-h period. *Pst* DC3000 (pHW01) cells were alive and did not fluoresce in the absence of leaves until the completion of the experiment (**Figure 4A**). Strong green fluorescence was evident in several attached bacteria (2.42 ± 0.36 bacteria per 0.075 mm^2^) at approximately 3–4 h after contact with the surface of intact leaf (**Figure 4B**). At 24 h, we observed that all bacterial cells on the entire leaf surface were fluorescing; a representative micrograph is shown in **Figure 4B**. Bacteria in suspension exposed to the leaf surface were also fluorescing at the same time points as the attached cells (**Figure 4C**), indicating that bacterial attachment is not required for the induction of COR biosynthesis.

**FIGURE 5:**
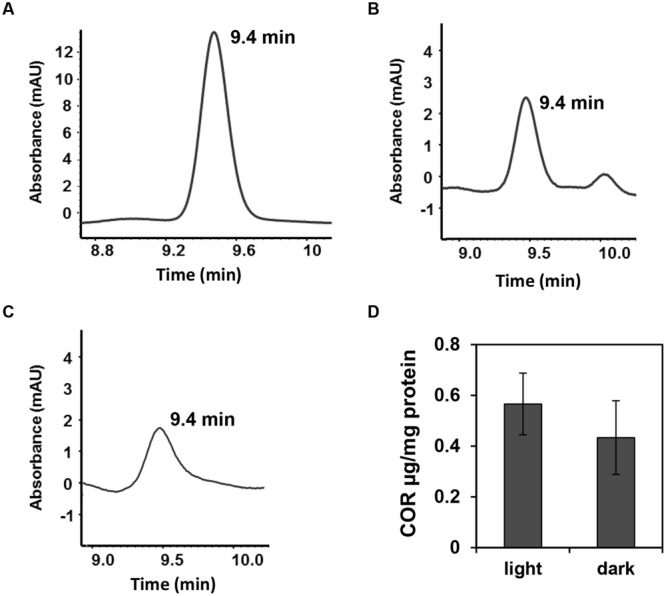
***Pst* DC3000 produces comparable amounts of COR under light or darkness.** *Pst* DC3000 was grown in COR inducing medium (HSC medium) for 24 h in constant light (70–80 μmol.m^-2^.s^-1^) or constant darkness and COR production was assessed by HPLC. **(A–C)** Chromatograms obtained by HPLC showing peaks of COR at the retention time of 9.4 min when the sample injected was 15 μg.ml^-1^ COR (control) **(A)**, *Pst* DC3000 grown in light **(B)** or *Pst* DC3000 grown in dark **(C)**. mAU; milliAbsorbance Units at 208 nm. **(D)** COR concentration in *Pst* DC300 cells grown in light or dark calculated as μg COR per mg of total protein. Data points are shown as mean (*n* = 6) ± SE and no statistical significance was observed between the means.

To assess whether light is required or not for the induction of the *cma* promoter in epiphytic *Pst* DC3000, the same experiment reported in **Figure 4** was repeated under constant darkness. Bacterial suspension in contact with the leaf surface was examined for green fluorescence up to 24 h of exposure to leaves. We observed the same activation pattern of the *cma* operon in the dark (data not shown) indicating that COR biosynthesis is independent of the light regime. Using an HPLC-based method, we also confirmed that light regimes have no effect on COR production by *Pst* DC3000 cultured in COR-inducing medium. COR was detected in the supernatants of bacterial cultures grown in the presence or absence of white light as evidenced by an absorbance peak at the 9.4 min retention time, similar to the absorbance profile obtained with the injection of pure COR solution (**Figures 5A–C**). The amount of COR produced by *Pst* DC3000 was very similar between the two culturing conditions (**Figure 5D**).

## Discussion

It is well documented in the literature that environmental conditions significantly affect the interactions between plants and pathogens. In this study, we demonstrate that darkness induces a pronounced decrease in the stomatal aperture width of *Arabidopsis*, which contributed to diminishing bacterium penetration in the leaf apoplast as most of the stomatal pores are closed. In this scenario, fewer bacterial cells would be able to freely invade the leaf interior, consequently decreasing the severity of diseases. Therefore, in the absence of extensive wounding, epiphytic bacteria may have to infect leaves primarily during the day when most stomata are opened and bacterium-induced stomatal closure is not as pronounced as in darkness (**Figure 3B**), or employ virulence strategies to overcome stomatal immunity.

Some highly virulent bacterial pathogens secrete small molecules that open the stomatal pore (Melotto et al., 2006; Gudesblat et al., 2009; Schellenberg et al., 2010). For instance, oxalic acid produced by *Sclerotinia sclerotiorum* and COR produced by *P. syringae* have been shown to induce stomatal opening in leaves of *Vicia faba* (Guimarães and Stotz, 2004), broad bean and Italian ryegrass (Mino et al., 1987) in the dark. However, this phenomenon has not been linked to plant infection by these toxin-producing pathogens.

Taking advantage of the *Arabidopsis*–*Pseudomonas* pathosystem, we were able to provide pharmacological and genetic evidence that COR not only blocks bacterium/PAMP-triggered stomata closure (**Figure 1**; Melotto et al., 2006), but also promotes opening of stomata at night altering the circadian stomatal movement (**Figure 2**). The molecular mechanisms underlying stomatal closure and opening are not completely overlapping (Yin et al., 2013), which suggests that COR may affect multiple pathways in the guard cell. It is not yet known, whether COR opens dark-closed stomata using the same pathway components involved in light-induced stomatal opening. At this moment, COR has only been shown to modulate ion channels in the guard cell plasma membrane; it reverses PAMP-inhibition of K^+^_in_ currents resulting in stomatal opening (Zhang et al., 2008). Nonetheless, our results indicate that COR interferes directly or indirectly with circadian regulation of stomatal movement. Furthermore, dark had a greater effect on the apoplastic population size of the COR-mutant *Pst* DC3118 as compared to the COR-producing *Pst* DC3000 in the first day after inoculation (**Figure 3C**). Altogether, these results suggest that COR can promote bacterial entry in the dark, which may provide an epidemiological advantage to COR-producing *P. syringae* over non-COR-producing strains by invading plants during the night, a condition that naturally favors stomatal-based defenses.

It is important to note that similar to what have been observed in tomato plants (Ishiga et al., 2009), we also found that *Arabidopsis* plants inoculated with *Pst* DC3000 under dark has less pronounced disease symptoms than plants kept under a 12 h photoperiod (**Figure 3D**). These results suggest a distinct, light-dependent role of COR in the late stages of the disease and highlight the multifaceted functions of COR throughout the life cycle of *P. syringae*.

*Pst* DC3000 possesses a two-component system that regulates the production of COR at the transcriptional level in *P. syringae* (Braun et al., 2009). COR is formed by a link between coronafacic acid (CFA) and coronamic acid (CMA) and the genes required for the biosynthesis of these COR precursors are organized into two operons, *cfl/cfa* and *cma* (Budde et al., 1998). These COR biosynthesis genes have been found to be expressed only when the bacterium is cultured in inducing minimum medium or *in planta* after a few hours of inoculation (Palmer and Bender, 1993; Li et al., 1998; Boch et al., 2002; Braun et al., 2009). For instance, the *Pst* DC3000 *cfl/cfa* operon has been shown to be induced 6 h after bacterial infiltration into *Arabidopsis* leaves (Boch et al., 2002). Similarly, the *cma* promoter of *P. syringae* pv. *glycinea* PG4180 is strongly activated inside of soybean leaves within 6 h after bacterial infiltration (Braun et al., 2009). To the best of our knowledge, for the first time our results provide evidence that COR biosynthetic gene expression is induced in the epiphytic phase of the pathogen. Additionally, our results demonstrate that induction of COR biosynthesis requires contact with the leaf cuticle and it can occur prior to bacterial penetration through the stomata, independent of the light condition.

The use of the reporter strain *Pst* DC3000 pHW01 allowed the interesting observation that COR biosynthesis can be induced in isolated cells attached to or in a suspension in contact with the leaf surface (**Figure 4**). In agreement with this result, Li et al. (1998) reported that bacterial population sizes in various plants did not correlate with the reporter gene activity using a *cor:inaZ* marker-exchange strain of DC3000. Thus, it is tempting to speculate that COR biosynthesis may not depend on or it can play a role upstream of cell-cell communication, such as *Pst* DC3000 quorum sensing activities previously documented by Chatterjee et al. (2007).

The induction and production of COR by *Pst* DC3000 under both light and darkness provided further evidence for the ability of this bacterium to penetrate leaves at night. *Pst* DC3000 aggregates and moves toward open stomata (Melotto et al., 2006); however, light conditions itself can affect pathogen virulence by regulating motility. For instance, *Pst* DC3000 perceives light through its photoreceptors, and exposing bacterial cultures to white light prior to inoculation has been shown to inhibit motility and promote attachment of bacteria on the leaf surface (Río-Álvarez et al., 2014). Although the involvement of COR in epiphytic fitness is not investigated here, the possibility of COR helping epiphytic survival of *Pst* DC3000, in addition to mediating stomatal opening, cannot be ruled out. Indeed, the involvement of virulence factors, such as the phytotoxin mangotoxin, type III secretion system components, and the exopolysaccharide alginate, in the epiphytic fitness of *P. syringae* pv. *syringae* has been described (Yu et al., 1999; Arrebola et al., 2009; Lee et al., 2012). In the future, it would be important to identify the environmental signal(s) that control COR production on the leaf and to investigate whether COR mediates additional aspects of bacterial interactions with the plant surface.

## Author Contributions

MM conceived and designed research; SP, DR, RC, LP, ZB, and MM performed research; DA and MM contributed material/analytic tools; SP, ZB, and MM analyzed data; and SP and MM wrote the paper. All authors have read and approved the final version of the manuscript.

## Conflict of Interest Statement

The authors declare that the research was conducted in the absence of any commercial or financial relationships that could be construed as a potential conflict of interest.
